# Combining trauma therapy with computer-based neurocognitive training in soldiers with post-traumatic stress disorder: a quasi-experimental study on symptom reduction and long-term memory functions

**DOI:** 10.3389/fpsyt.2026.1750423

**Published:** 2026-02-18

**Authors:** Patric Muschner, Lena Goldschmidt, Luisa Sophie Gröning, Jennifer Spohrs, Gerd-Dieter Willmund, Martina Piefke

**Affiliations:** 1 Neurobiology and Genetics of Behavior, Department of Psychology and Psychotherapy, Witten/Herdecke University, Witten, Germany; 2 Clinic of Psychiatry, Psychotherapy and Psychotraumatology, Bundeswehr Hospital, Ulm, Germany; 3 Bundeswehr Centre for Military Mental Health, Bundeswehr Hospital Berlin, Berlin, Germany

**Keywords:** military personnel, neurocognitive deficits, neuropsychological intervention, psychotherapeutic trauma treatment, PTSD, verbal memory, visuospatial memory

## Abstract

**Introduction:**

Cognitive training has the potential to reduce symptoms of Post-Traumatic Stress Disorder (PTSD), but its effects on memory dysfunctions related to the disorder remain unclear. Goal of this study was to examine if a computer-based neurocognitive training (c-bnt) improves verbal and visuospatial long-term memory and reduces PTSD and depressive symptoms in active soldiers with PTSD.

**Methods:**

In a quasi-randomized controlled design, soldiers suffering from PTSD were assigned to an intervention group (IG) or treatment as usual group (tauG). Long-term memory performance was assessed using the Verbal Learning and Memory Test (VLMT) and Visual Path Learning Test (VWLT) at baseline and after a three-week intervention period. Severity of PTSD symptoms and depression was assessed with the *PTSD Checklist for DSM 5* (PCL-5) and the revised version of Beck’s Depression Inventory (BDI-II). Mixed ANOVAs and mediation analyses were applied to test intervention effects and putatively mediating influences of depression.

**Results:**

Compared to the tauG, the IG showed significant reductions in the PTSD symptoms avoidance (*p* = .038), negative cognitions (*p* = .044), as well as depression (*p* = .026). No significant improvements in verbal or visuospatial long-term memory were observed (all *p* >.05). Mediation analyses revealed no indirect effects of depressive symptoms on memory outcomes.

**Discussion and Conclusion:**

C-bnt was associated with modest, domain-specific reductions in PTSD-related avoidance and depressive symptoms at post-treatment, while no improvements in verbal or visuospatial long-term memory performance were observed. Symptom relief may thus be based on intermingled changes of diverse cognitive resources and emotional processes. We suggest that c-bnt may serve as a low-threshold adjunct to trauma-focused therapy, that may help to improve health condition and capabilities of everyday life in soldiers with PTSD.

## 1 Introduction

Post-traumatic stress disorder (PTSD) is a mental disorder, with a range of distressing symptoms, such as re-experiencing of the event, avoidance behavior, increased arousal and/or negative changes in mood or cognition, that frequently results after the experience of traumatic events (1). It is associated with a high risk of comorbid conditions, such as depression, anxiety and/or substance use disorder (SUD) (2–5). While one-month prevalence of PTSD in Germany ranges between 1.3–1.9% for civilians under 60 years of age (3), a significant increase in the risk of PTSD was observed in studies based on samples of deployed soldiers with combat experience (6, 7), with some studies even indicating an up to fourfold increased risk of illness (8). A twelve-month prevalence of up to 2.9% was found, although this likely underestimates the actual occurrence due to high number of unidentified and untreated PTSD cases (8). One resulting negative impact apart from the general symptoms of PTSD is its negative effects on the patient’s ability to work due to illness-related functional limitations (9–12).

PTSD is associated with various biological and neurological changes, which in turn can lead to impaired cognitive performance. For example, morphological and/or functional abnormalities in brain regions such as the amygdala, hippocampus and prefrontal cortex have been observed in patients with PTSD (13–16). Further, negative effects of PTSD on memory functions are well documented, especially for long-term memory (17, 18).

Similar cognitive impairments have been identified in patients with depression (19–24). The common memory impairments include auditory-verbal and visual memory. Since a large number of soldiers with PTSD, up to 48% (25), also suffer from a comorbid depressive disorder, memory decline may arise from either disorder.

Meta-analyses have demonstrated that both verbal learning and memory (e.g., immediate and delayed recall) as well as visuospatial memory are affected, with medium to large effect sizes compared to healthy controls (26, 27). These impairments are thought to be related to altered functioning in brain areas such as the hippocampus and the prefrontal cortex, which are frequently implicated in PTSD (28). Importantly, memory problems are not limited to trauma-related content but extend to neutral information as well (29). While verbal memory difficulties have been associated with intrusions and dissociation, visuospatial memory seems to be particularly vulnerable to distraction and emotional interference (30). Recent systematic evidence provides a more comprehensive overview of these cognitive intervention approaches. A meta-analysis by Muschner, Goldschmidt (31) summarized eight randomized controlled trials on computer-based neurocognitive training (c-bnt) in PTSD and comorbid depression. Although the pooled effect on PTSD symptom severity was small and statistically non-significant (*d* = −0.21, *p* = .31), the authors observed indications of improvements in working memory and cognitive flexibility. These results highlight both the potential and the current methodological limitations of c-bnt approaches and underscore the need for studies addressing broader memory domains. Despite this, there are only few clinical studies that have tested interventions targeting memory performance in PTSD, and even fewer that have considered both verbal and visuospatial memory in the same design (32, 33).

In recent years, studies combining basic research and clinical approaches, as well as military-relevant investigations have reinforced the evidence for broad memory deficits in PTSD (34–36). Specifically, visuospatial working memory and episodic memory are consistently reported to be impaired across studies. Experimental interventions such as Tetris-based visuospatial interference tasks have shown mixed results in reducing intrusive memories. Recently, training tools such as Mobilum have been developed, aiming to provide an alternative to Tetris-based interventions (37). Mobilum is an Android-based visuospatial training app involving the mental rotation of complex 3D figures. While it has been proposed as a practical substitute, empirical evidence on its clinical utility is currently limited (37).

Cognitive training approaches have increasingly been evaluated in the treatment of PTSD because of their assumed potential to reduce PTSD symptoms indirectly by improving cognitive functions such as attention and working memory. Evidence for this argument comes from studies on attention bias modification (38, 39). Although these studies rarely demonstrated robust improvements in trained cognitive functions, the authors reported moderate reductions in PTSD symptom severity. This suggests that cognitive interventions may exert psychotherapeutic benefits through intermingled mechanisms including improved integration of cognitive and emotional processes, rather than direct enhancement of single cognitive basic functions.

From a military perspective, cognitive training approaches may offer specific advantages over traditional trauma-focused psychotherapy. Numerous studies have shown that active-duty soldiers and veterans often face substantial barriers against engaging in psychotherapy. These include stigma, concerns about career impact, and limited availability of trauma-informed services during or after deployment (40, 41). Even when mental health symptoms are present, service members may postpone or avoid treatment due to fear of appearing weak, institutional mistrust, or concerns about operational readiness (40, 41). In this context, low-threshold, task-oriented cognitive interventions may represent a better acceptable alternative. Because they are shaped as performance-enhancing rather than emotionally confronting, such interventions may reduce help-seeking resistance and increase compliance (42). Moreover, even moderate reductions in PTSD symptoms, particularly in entangled cognitive and affective domains such as avoidance or negative cognitions, may improve soldiers’ occupational functioning, social reintegration, and fitness for duty. This makes cognitive training particularly relevant in military or paramilitary populations.

Despite their practical relevance, few studies have examined whether cognitive training improves long-term memory in PTSD. A meta-analysis by Sep, Geuze (35) confirmed consistent impairments in learning and memory across both clinical and preclinical PTSD samples. However, direct evidence from training studies remains limited. One pilot RCT by Moradi, Moshirpanahi (33) applied Memory Specificity Training (MEST), a structured intervention aimed at improving access to specific autobiographical memories by reducing overgeneral memory retrieval. They found improved autobiographical memory and reduced PTSD symptoms in combat veterans. Moreover, Bomyea, Caudle (32) tested a computerized working memory training in U.S. veterans and reported moderate reductions in re-experiencing symptoms (Hedges’ *g* = 0.57). While these findings point to symptom relief through memory-related mechanisms, broader trials assessing long-term memory outcomes are still lacking.

Long-term memory processes are centrally involved in the development and maintenance of trauma-related disorders. Intrusive re-experiencing, impaired extinction learning, and fragmented autobiographical recall have all been linked to dysfunctional memory encoding and retrieval. Verbal long-term memory dysfunctions have been associated with dissociative symptoms and re-experiencing (29), while visuospatial long-term memory is particularly vulnerable to distraction, interference, and emotional dysregulation (30, 34). Cognitive interventions that specifically target both forms of long-term memory may thus be beneficial in symptom reduction. Although long-term memory impairments are well-documented features of PTSD (35), they rarely occur in isolation. Long-term memory performance depends on cognitive control processes such as directing attention, inhibition of interference, as well as online processing of information in working memory - Neuropsychological functions, which are also frequently disrupted in individuals with PTSD. A recent systematic review by Susanty, Sijbrandij (43) found that trauma-centered psychotherapeutic interventions tend to enhance memory performance more consistently than executive functions or attention. This finding suggests that memory functions may indirectly benefit via broader cognitive and emotional systems. Moreover, experimental studies have demonstrated that even when cognitive interventions do not directly train long-term memory components, such as verbal or visuospatial recall, they may lead to symptom reduction. For example, Schweizer, Grahn (44) showed that training of emotional working memory improved affective control and reduced PTSD symptoms. These findings suggest that interventions may exert indirect effects on memory-related symptoms via improved emotion regulation or executive control, rather than through direct enhancement of long-term memory structures. Based on this evidence, our approach focuses on multimodal training targeting interrelated domains to enhance multiple functions that support and modulate memory processes.

The present study investigates whether the addition of c-bnt to trauma-focused therapy as usual (tauG) may improve both verbal and visual-spatial long-term memory and reduce PTSD and depressive symptoms more efficiently than trauma focused therapy alone. Given the role of long-term memory dysfunctions in PTSD, we hypothesize that participants in the IG would show greater improvements in memory functions and PTSD symptoms than tauG. Furthermore, we expect that the severity of PTSD and depressive symptoms would negatively affect training outcomes on memory, and that depressive symptomatology acts a mediator of changes of both PTSD symptoms and verbal and visuospatial long-term memory.

## 2 Methods

### 2.1 Participants

Participants were male soldiers with a deployment-related PTSD. Female participants were not included by methodological design consideration, as the study involved the assessment of biological stress markers (e.g., cortisol), and menstrual cycle-related hormonal variability would have introduced additional potential bias. Soldiers were included into the study if they met the following inclusion criteria: (1) age > 18 years, (2) diagnosed PTSD, and (3) fluency in written and spoken German language, as well as unimpaired vision and hearing. Exclusion criteria were: (1) comorbid psychosis, neurological illnesses, and severe medical disorders, (2) the use of highly potent neuroleptics, and (3) acute suicidality. Comorbid depression, anxiety disorder, and adjustment disorder were no exclusion criteria. The soldiers were recruited at the military hospitals in Berlin and Ulm. All study participants received inpatient treatment with trauma-focused therapy in military hospitals. The study was approved by the ethics committee of Witten/Herdecke University (Application No. 196/2016) and is in compliance with the Declaration of Helsinki. A total of *N* = 53 participants were included in the study. Eligible soldiers were identified and invited to participate in the context of routine clinical care. Only soldiers who provided written informed consent for study participation were included systematic documentation. The total number of potentially eligible soldiers who were approached but declined participation was not recorded; therefore, a formal participation rate cannot be reported. In accordance with German data protection regulations, documentation of potential participants was only permitted after written informed consent had been obtained.

#### 2.1.1 Baseline group differences in sociodemographic characteristics

Group differences in sociodemographic variables were examined using independent-samples *t*-tests for continuous variables. There were no statistically significant differences between the groups for age (*t*(33.87) = –0.07, *p* = .949, *d* = –0.02), number of children (*t*(32) = 0.39, *p* = .695, *d* = 0.14), net monthly income (*t*(40) = 1.01, *p* = .320, *d* = 0.31), disposable income (*t*(20.16) = 1.25, *p* = .225, *d* = 0.45), years of service (*t*(14) = –2.15, *p* = .050, *d* = –1.08), total days deployed (*t*(44) = 1.20, *p* = .235, *d* = 0.36), duration of the last assignment abroad (*t*(42) = 0.03, *p* = .980, *d* = 0.01), or estimated intelligence quotient (*t*(50) = 0.66, *p* = .510, *d* = 0.18). However, a statistically significant difference was found in the number of deployments, with IG reporting more deployments (*t*(24.36) = 2.16, *p* = .041, *d* = 0.69). This represents moderate effect sizes.

Descriptive statistics and group comparisons for all sociodemographic and service-related variables are presented in **Table 1**.

**Table 1:** Baseline group comparisons of sociodemographic variables at t_1_.

Variable	TS *M* (*SD*)	IG *M* (*SD*)	tauG *M* (*SD*)	*p*	Cohen´s *d*
Age	39.98 (8.51)	41.35 (9.91)	41.50 (5.62)	.949	-0.02
Number of children	2.2 (1.22)	2.27 (1.34)	2.11 (1.05)	.695	0.14
Net monthly income	4261.59 (1578.63)	4566.67 (2019.32)	4092.42 (978.63)	.320	0.31
Disposable income	1408.51 (1006.73)	1688.24 (1468.79)	1214.58 (626.50)	.225	0.45
Years of service	15.14 (5.70)	12.33 (6.14)	18.14 (4.1)	.050	-1.08
Number of deployments	5.06 (4.18)	6.90 (5.54)	4.15 (2.03)	.041*	0.69
Total days deployed	607.35 (433.09)	735.55 (551.93)	584.81 (283.31)	.235	0.36
Last deployment duration in days	127.23 (67.44)	134.74 (68.09)	134.24 (62.23)	.980	0.01
Estimated IQ	103.53 (7.93)	104.22 (7.73)	102.71 (8.67)	.510	0.18

TS, Total sample; IG, Intervention Group; tauG , treatment as usual Group; *p <.05 (two-tailed); Cohen’s d represents standardized mean differences between the intervention group and the treatment as usual group.

### 2.2 Design and procedure

**Figure 1:**
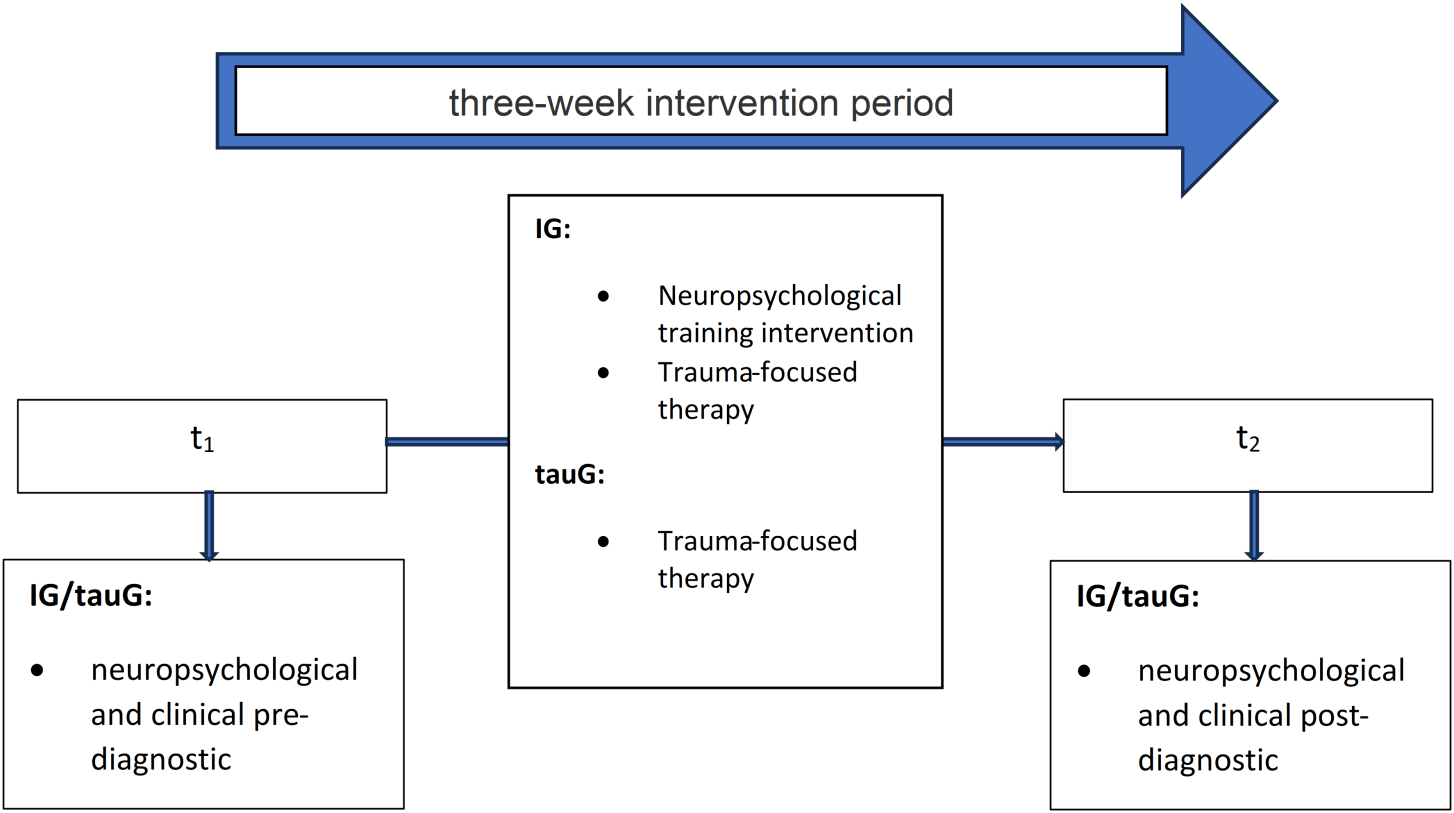
Study design illustrating the quasi-randomized assignment of participants to either the IG or tauG. Each group received inpatient trauma-focused therapy. The IG additionally accomplished c-bnt. Assessments took place at baseline (t_1_) and post-intervention (t_2_).

In this study with group and pre- and post-intervention comparisons, soldiers with PTSD were quasi-randomly assigned to one of two groups, which does not fully control for expectancy effects or pre-existing group differences. The IG received an app-based neuropsychological training developed by the company *HeadApp* (Gommern, Germany; https://www.headapp.com/de/startseite-2-2/). The c-bnt program comprised modules targeting attentional control, working memory updating, planning, and cognitive flexibility. These processes were hypothesized to indirectly support long-term verbal and visuospatial memory performance by facilitating efficient encoding, strategic organization, and executive monitoring during learning and retrieval. However, the intervention was not specifically designed to train long-term verbal or visuospatial memory functions, and transfer to standardized long-term memory measures was therefore considered exploratory. The tauG was a treatment as usual group treated with trauma therapy only. All participants in both groups received inpatient trauma-focused treatment as usual (TAU) at German military hospitals. TAU was delivered by licensed trauma therapists and comprised different evidence-based trauma-focused approaches, including Eye Movement Desensitization and Reprocessing (EMDR), Imagery Rescripting and Reprocessing Therapy (IRRT), and Cognitive Processing Therapy (CPT). Trauma-focused psychotherapy was typically delivered at a frequency of approximately two sessions per week, consistent with standard inpatient military care. In line with standard inpatient military care, TAU followed a multimodal treatment approach and additionally included complementary therapeutic elements (e.g., psychoeducation, physical activity, and supportive interventions), which were equally available to both groups. Assignment of patients to therapists occurred randomly as part of routine clinical care. Treatment was not manualized within the framework of the present study, and specific therapeutic modalities were not documented at the individual level. All participants underwent a comprehensive clinical and neuropsychological examination at baseline (t_1_) and after three weeks of therapy (t_2_). The study design is illustrated in **Figure 1**. Of the *N* = 53 participants assessed at baseline (t_1_), *n* = 34 completed the post-intervention assessment at t_2_, resulting in an attrition rate of 35.85%.

### 2.3 Materials

#### 2.3.1 Clinical inventories

Potential participants were identified prior to admission based on CAPS-5-Interviews an existing clinical diagnosis of PTSD for inpatient trauma-focused treatment. After written informed consent was obtained, PTSD diagnosis was formally confirmed by the study team using the *Diagnostic Interview for Mental Disorders* (Mini-DIPS) (45). The *PTSD Checklist for DSM 5* (PCL-5) was used exclusively to assess symptom severity and was not applied for diagnostic purposes (46, 47). The accompanying *Life Events Checklist for DSM 5* (LEC-5) assessed trauma exposure, including whether soldiers experienced the event(s) firsthand and/or as witnesses. The revised *Beck Depression Inventory* (BDI-II) was used to identify comorbid depressive disorders (48).

#### 2.3.2 Neuropsychological assessment

At t_1_ and t_2_, all study participants were assessed neuropsychologically with a comprehensive testing battery that targeted memory functions, but also executive functions, attention, and social cognition. In this paper, we report data on both verbal and visual-spatial long-term memory, assessed with the *Verbal Learning and Memory Test* (VLMT (49)) and the *Visual Path Learning Test* (VWLT) (50).

#### 2.3.3 C-bnt

The *Headapp* platform at https://www.headapp.com (HeadApp, Gommern, Germany) was used for the computer-based neuropsychological intervention. It involved a daily one-hour neuropsychological training program comprising three 20-minute sessions of the modules *Flip It*, *Vita Plan*, and *Vita Att*, each targeting distinct neuropsychological functions. The order of modules execution was automatically changed each training day.

**Figure 2:**
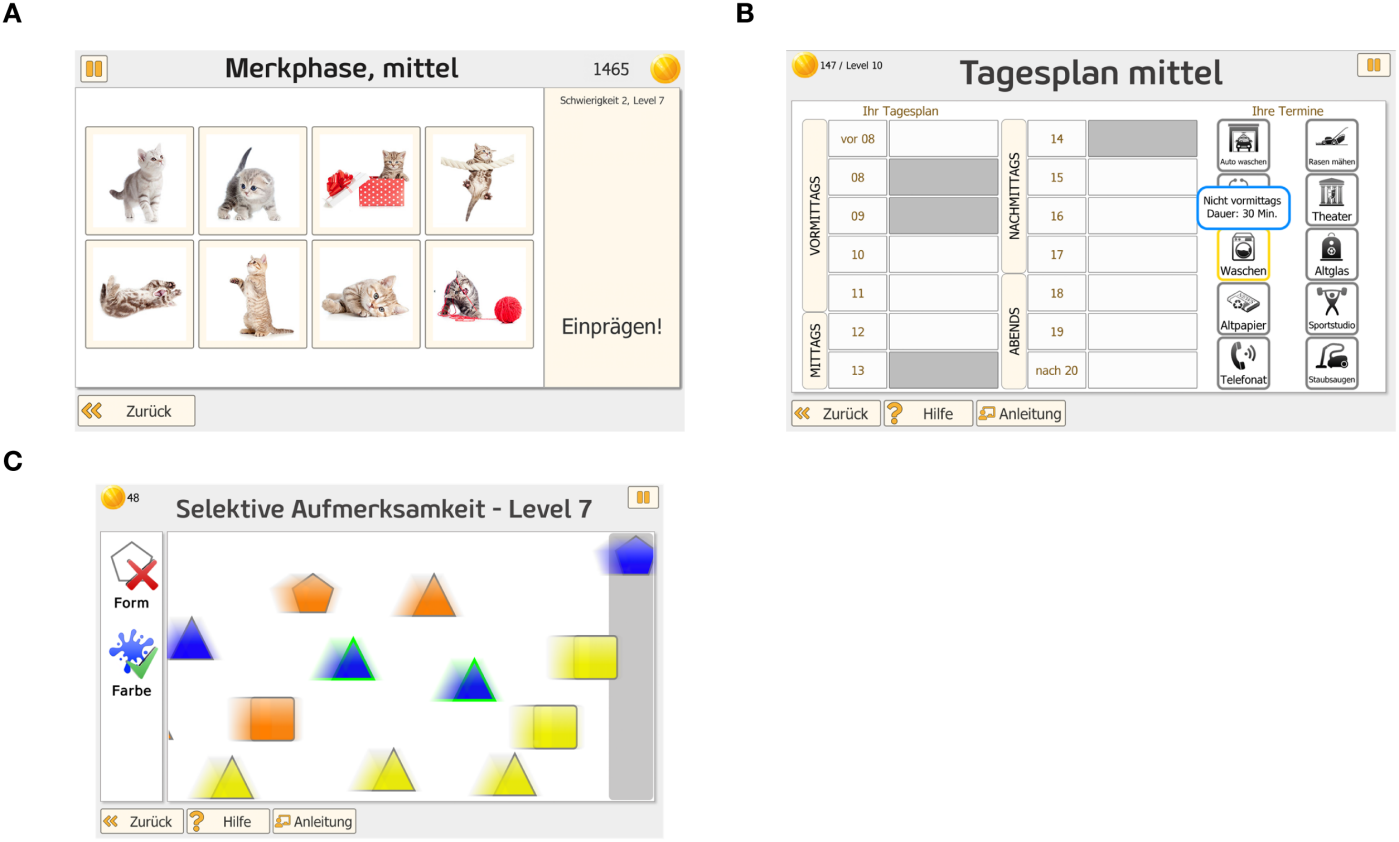
**(a)***Flip it*. Example screen view of the *Flip It* module of the c-bnt. The task trains short- and long-term memory by requiring participants to memorize the location of images or words on the screen and recall them after brief masking. Performance is reinforced by a coin-based feedback system and level progression indicators. **(b)***Vita Plan*. Example screen view of the Vita Plan module of the c-bnt. The task targets planning and problem-solving by requiring participants to arrange a set of appointments in chronological order based on time slots. Correct solutions are rewarded with coins, and performance progress is tracked via a colored progress bar. **(c)***Vita Att*. Example screen view of the Vita Att module of the c-bnt. The task targets attentional control and cognitive flexibility by requiring participants to respond to moving geometric shapes according to changing rules. Correct responses are rewarded with coins, and difficulty increases progressively across levels.

The *Flip It* task trains short-term and long-term memory and is based on the classic “memory” game (see **Figure 2a**). Subjects are asked to remember displayed pictures or words together with their position on the screen. Once these are covered, the target object (word or picture) is presented and the subjects are required to memorize its position among the covered boxes. For each correct memorization, the subject gains a number of coins, while each wrong guess leads to a loss of a number of coins. Progress in each level is indicated by stars at the top of the screen. When the subject reaches optimum performance in one level of the game, he moves on to a new and more difficult level of the module.

The *Vita Plan* module targets planning and problem-solving. Subjects are required to arrange displayed appointments in chronological order based on the time frames displayed above each appointment (see **Figure 2b**). The appointments are then dragged and dropped into the appropriate time slot. Once all appointments are sorted, the results are logged and reviewed. Correct answers are rewarded with coins, while incorrect assignments result in loss of coins. Progress within the module is indicated by a yellow/green progress bar showing level advancement.

*Note*. Example screen view of the *Vita Plan* module of the c-bnt. The task targets planning and problem-solving by requiring participants to arrange a set of appointments in chronological order based on time slots. Correct solutions are rewarded with coins, and performance progress is tracked via a colored progress bar.

The *Vita Att* task targets attention and switching flexibility (see **Figure 2c**). Participants must click on geometric figures moving from left to right before reaching the edge of the screen, based on changing rules. Correct selections increase the coin count while errors lead to loss of coins. Progress in the game is indicated by a yellow/green bar. The current coin count and reached level are displayed in the upper left corner of the screen. The rules’ difficulty and complexity increase with each level.

*Note*. Example screen view of the *Vita Att* module of the c-bnt. The task targets attentional control and cognitive flexibility by requiring participants to respond to moving geometric shapes according to changing rules. Correct responses are rewarded with coins, and difficulty increases progressively across levels.

#### 2.3.4 Statistical analyses

An *a priori* sample size calculation was conducted to detect a between-group difference of one scale point in pre–post change scores (primary endpoint), assuming *α* = .05 and 80% power. Based on two parallel independent *t*-tests comparing the combination condition versus treatment as usual, the required sample size for the PTSD stratum was *N* = 2 × 37 participants. All statistical analyses were conducted using IBM SPSS Statistics Version 29 (51). Mediation models were calculated using the PROCESS macro for SPSS (Model 4) (52) and were conducted in an exploratory, hypothesis-generating framework without assumptions of causal inference. Descriptive statistics were computed for all variables. To examine baseline group differences in sociodemographic, clinical, and cognitive characteristics, independent-samples *t*-tests were performed. Hypotheses were tested using mixed-design ANOVAs. In addition, group differences at post-assessment (t_2_) were examined using one-tailed *t*-tests based on *a priori* directional hypotheses. These post-treatment group comparisons were conducted within an exploratory analytic framework and were not intended as confirmatory tests. In the absence of significant group × time interaction effects, post-assessment comparisons were interpreted descriptively and as hypothesis-generating rather than as evidence of differential treatment effects. Potential influences of depressive symptoms on the relationship between PTSD severity and memory performance were examined via correlation and mediation analyses. All statistical tests were conducted at a significance threshold of *α* = .05 (two-tailed unless stated otherwise), and confidence intervals (CI) were set at 95%. No adjustment for multiple comparisons was applied. Given the exploratory nature of the study, the limited sample size, and the focus on detecting potential patterns rather than confirmatory hypothesis testing, applying conservative familywise error rate corrections (e.g., Bonferroni) or false discovery rate procedures was considered to be inappropriate, as this would have further reduced statistical power and increased the risk of type II errors.

## 3 Results

For clarity, the Results section emphasizes predefined primary clinical and neurocognitive outcomes. Detailed null findings and exploratory analyses are reported for transparency but should be interpreted in the context of the study’s exploratory design and limited statistical power.

### 3.1 Primary clinical outcomes (PCL-5 total, subscales; BDI-II)

Independent samples *t*-tests at t_1_ revealed no significant baseline differences between the intervention and treatment as usual groups on any PCL subscale (see **Table 2a**): Intrusion (*t*(42) = 0.63, *p* = .529, *d* = 0.19), Avoidance (*t*(42) = –0.08, *p* = .936, d = 0.02), Negative Cognitions (*t*(42) = –0.84, *p* = .408, *d* = 0.25), Arousal (*t*(42) = –0.08, *p* = .935, *d* = 0.03), and overall PTSD symptom severity (*t*(42) = –0.22, *p* = .831, *d* = 0.07). There was also no significant baseline difference in depressive symptom severity (BDI-II) between the IG and tauG (*t*(41) = –1.07, *p* = .291, *d* = 0.327).

**Table 2a:** Descriptive statistics of clinical measures at t_1_.

Variable	IG *M* (*SD*) [*n*]	tauG *M* (*SD*) [*n*]	*p*_(two-tailed)_
PCL Intrusion	12.83 (4.47) [23]	11.90 (5.17) [21]	.529
PCL Avoidance	5.61 (1.90) [23]	5.67 (2.82) [21]	.936
PCL Negative Cognitions	14.74 (7.20) [23]	16.57 (7.33) [21]	.408
PCL Arousal	14.74 (4.56) [23]	14.86 (4.99) [21]	.935
PCL Total	47.91 (16.79) [23]	49.00 (16.68) [21]	.831
BDI-II	23.91 (9.38) [23]	27.45 (12.27) [20]	.291

IG, Intervention Group; tauG, treatment as usual Group; *M*, mean, *SD*, standard deviation; PCL and BDI-II values represent raw scores. Values reflect pre-treatment group comparisons using independent-samples *t*-tests (*p <.05).

Independent-samples *t*-tests were conducted to examine whether post-treatment (t_2_) symptom levels were lower in the IG compared to the tauG (see **Table 2b**). Significant group differences emerged for Avoidance (*t*(32) = –1.84, *p* = .038, *d* = 0.63), Negative Cognitions (*t*(32) = –1.76, *p* = .044, *d* = 0.60), and depressive symptoms (*t*(32) = –2.03, *p* = .026, *d* = 0.69), all indicating moderate effect sizes. No significant differences were found for Intrusion (*p* = .283, *d* = 0.32), Arousal (*p* = .094, *d* = 0.47), or overall PTSD severity (*p* = .074, *d* = 0.49), though small to moderate effects were observed.

**Table 2b:** Descriptive statistics of clinical measures at t_2_.

Variable	IG *M* (*SD*) [*n*]	tauG *M* (*SD*) [*n*]	*p*_(one-tailed)_
PCL Intrusion	12.59 (5.93) [17]	13.59 (3.92) [17]	.283
PCL Avoidance	4.64 (2.57) [17]	6.18 (2.27) [17]	.038*
PCL Negative Cognitions	14.35 (8.71) [17]	18.82 (5.85) [17]	.044*
PCL Arousal	14.06 (5.89) [17]	16.29 (3.42) [17]	.094
PCL Total	45.65 (21.99) [17]	54.88 (12.86) [17]	.074
BDI-II	22.35 (14.59) [17]	31.76 (12.40) [17]	.026*

IG, Intervention Group; tauG, treatment as usual Group; *M*, mean; *SD*, standard deviation; PCL and BDI-II values represent raw scores. Values reflect post-treatment (t_2_) group comparisons using independent-samples *t*-tests (*p <.05).

### 3.2 Effects of the intervention on PTSD and depressive symptoms

All statistical assumptions (normality, homogeneity of variance, and equality of covariance matrices) were assessed and considered in the interpretation of results. Mixed-design ANOVAs revealed no significant time × group interactions for any of the PTSD symptom domains: Intrusion, *F*(1, 30) = 0.70, *p* = .411, *η²* = .023; Avoidance, *F*(1, 30) = 0.42, *p* = .520, *η²* = .014; Negative Cognitions, *F*(1, 30) = 0.16, *p* = .688, *η²* = .005; and Arousal, *F*(1, 30) = 0.28, *p* = .603, *η²* = .009. Due to violations of variance homogeneity, the interaction effects for Arousal and overall PTSD symptom severity could not be interpreted.

Regarding the main effects of time, no significant changes in symptom severity were found across time points for Intrusion (*p* = .828), Negative Cognitions (*p* = 1.000), Arousal (*p* = 1.000), or total PTSD symptom severity (*p* = .753). A marginal trend was observed for Avoidance, *F*(1, 30) = 3.28, *p* = .080, *η²* = .098.

Similarly, no statistically significant main effects of group were observed for any domain: Intrusion, *F*(1, 30) = 0.09, *p* = .767, *η²* = .003; Avoidance, *F*(1, 30) = 3.66, *p* = .065, *η²* = .109; Negative Cognitions, *F*(1, 30) = 3.89, *p* = .058, *η²* = .115; Arousal, *F*(1, 30) = 1.49, *p* = .232, *η²* = .047; and total PTSD symptom severity, *F*(1, 30) = 2.29, *p* = .141, *η²* = .071. **Table 3** summarizes all statistical parameters of the mixed-ANOVAs for PTSD symptom clusters, including interaction, time, and group effects, along with associated effect sizes. Note that interaction effects for Arousal and total PTSD symptoms were not interpreted due to significant variance inequality.

**Table 3:** Mixed-design ANOVAs results for PTSD symptom domains and depressive symptoms: interaction, time, and group effects.

Variable	Interaction *F*	Interaction *p*	Interaction *η*²	Time *F*	Time *p*	Time *η*²	Group *F*	Group *p*	Group *η*²
Intrusion	0.70	.411	.02	0.05	.828	.00	0.09	.767	.00
Avoidance	0.42	.520	.01	3.28	.080	.10	3.66	.065	.11
Negative Cognitions	0.16	.688	.01	0.00	1.000	.00	3.89	.058	.12
Arousal	0.28	.603	.01	0.00	1.000	.00	1.49	.232	.05
PTSD total	0.19	.660	.01	0.10	.753	.00	2.29	.141	.07
BDI-II	0.06	.811	.00	0.17	.688	.00	3.44	.074	.11

*F*-values, *p*-values, and partial eta squared *(η*²) for interaction, time, and group effects are reported for each PTSD symptom cluster and depressive symptoms. Interaction effects for Arousal and PTSD total score were not interpreted due to significant variance inequality.

For the BDI II measures of depression, all statistical assumptions were met. A mixed-design ANOVA revealed no significant time × group interaction, *F*(1, 29) = 0.06, *p* = .811, *η²* = .002, indicating that changes in depressive symptoms over time did not differ between the intervention and control groups. The main effect of time was not significant, *F*(1, 29) = 0.17, *p* = .688, *η²* = .006, suggesting no overall change in depressive symptoms from pre- to post-assessment. The main effect of group was also not statistically significant, *F*(1, 29) = 3.44, *p* = .074, *η²* = .106, indicating no substantial overall difference in depressive symptom levels between groups.

### 3.3 Verbal and visuospatial learning and memory

Group comparisons concerning memory performance between the IG and the tauG at t_1_ independent samples *t*-tests revealed no significant baseline differences on any VLMT trial: A1–A5 (*t*(49) = 0.17, *p* = .869, *d* = 0.05), A6 (*t*(49) = –0.11, *p* = .915, *d* = –0.03), and A7 (*t*(49) = –0.82, *p* = .418, *d* = –0.23).

Similarly, no significant group differences at t_1_ were found on any VWLT subscale (see **Table 4a**): A1–A5, *t*(42) = 1.32, *p* = .193, *d* = 0.39; B, *t*(42) = 1.21, *p* = .233, *d* = 0.36; A6, *t*(42) = 1.97, *p* = .055, *d* = 0.60; A7, *t*(42) = 1.19, *p* = .240, *d* = 0.36; forgetting rate, *t*(42) = 0.70, *p* = .486, *d* = 0.21. Effect sizes were small to moderate, with the largest (yet nonsignificant) effect for short-delay recall (A6), indicating a trend toward better performance in the IG.

**Table 4a:** Descriptive statistics neuropsychological measures at t_1_.

Variable	IG *M* (*SD*) [*n*]	tauG *M* (*SD*) [*n*]	*p_(_*_two-tailed)_
VLMT A1–A5	52.12 (10.87) [25]	51.58 (12.46) [26]	.869
VLMT A6	11.00 (3.66) [25]	11.12 (4.01) [26]	.915
VLMT A7	10.32 (3.58) [25]	11.15 (3.71) [26]	.418
VWLT (A1–A5)	54.12 (11.44) [24]	49.50 (11.94) [20]	.193
VWLT (B)	58.13 (11.63) [24]	54.00 (10.76) [20]	.233
VWLT (A6)	54.21 (8.50) [24]	48.15 (11.85) [20]	.055
VWLT (A7)	49.21 (9.58) [24]	45.25 (12.43) [20]	.240
VWLT (A5–A7)	46.83 (10.77) [24]	44.65 (9.64) [20]	.486

IG, Intervention Group; tauG, treatment as usual Group; VLMT, Verbal Learning and Memory Test; A1–A5, learning trials; A6, immediate recall; A7, delayed recall; (A1–A5), Learning Performance; (B), Interference Performance; (A6), Short-Delay Recall; (A5–A7), Forgetting Rate; Values represent means (*M*) and standard deviations (*SD*). VLMT, values represent raw scores; VWLT, values represent age-normed T-scores (*M* = 50, *SD* = 10).

To assess potential group differences in verbal learning and memory performance at post-assessment (t_2_), independent samples *t*-tests were conducted for the VLMT subscales A1–A5, A6, and A7. No significant differences were found between the intervention and treatment as usual groups on any of the subscales for learning performance (A1–A5), *t*(37) = 0.18, *p* = .429, *d* = 0.06, short-delay- recall (A6), *t*(36.94) = −0.13, *p* = .450, *d* = −0.04, and long-delay recall (A7), *t*(32.72) = −0.63, *p* = .265, *d* = −0.20. Effect sizes were negligible to small across all subscales.

No significant group differences were found for learning performance (*t*(34) = 0.36, *p* = .357, *d* = 0.12), interference performance (*t*(34) = 0.16, *p* = .435, *d* = 0.06), short-delay recall (*t*(34) = 0.65, *p* = .261, *d* = 0.22), or long-delay recall (*t*(34) = 0.01, *p* = .495, *d* = 0.01). All effects were small and not statistically significant. However, for forgetting rate, a trend-level group difference emerged in favor of the tauG (*t*(34) = −1.56, *p* = .064, *d* = −0.52), indicating a lower forgetting rate in the tauG at t_2_. The IG, in contrast, showed a poorer outcome on this measure. The effect size was in the medium range, and the result approached significance in the expected direction. An overview of group differences at t_2_ is provided in **Table 4b**.

**Table 4b:** Descriptive statistics neuropsychological measures at t_2_.

Scale	IG *M* (*SD*)[*n*]	tauG *M* (*SD*)[*n*]	*p_(_*_one-tailed)_
VLMT A1–A5	54.67 (13.20) [21]	54.00 (9.24) [18]	.429
VLMT A6	11.52 (3.72) [21]	11.66 (3.31) [18]	.450
VLMT A7	11.90 (3.02) [21]	11.22 (3.72) [18]	.265
VWLT (A1–A5)	55.35 (10.58) [20]	53.81 (14.40) [16]	.357
VWLT (B)	54.65 (11.92) [20]	53.94 (13.75) [16]	.434
VWLT (A6)	52.60 (12.87) [20]	50.00 (10.79) [16]	.261
VWLT (A7)	49.80 (9.53) [20]	49.75 (11.80) [16]	.494
VWLT (A5–A7)	49.70 (9.80) [20]	54.31 (7.43) [16]	.064

IG, Intervention Group; tauG, treatment as usual Group; VLMT, Verbal Learning and Memory Test; A1–A5, learning trials; A6, immediate recall; A7, delayed recall; (A1–A5), Learning Performance; (B), Interference Performance; (A6), Short-Delay Recall; (A5–A7), Forgetting Rate; Values represent means (*M*) and standard deviations (*SD*). VLMT, values represent raw scores; VWLT, values represent age-normed T-scores (*M* = 50, *SD* = 10).

### 3.4 Effects of the intervention on learning and memory

Before conducting the mixed-design ANOVAs, assumptions were tested. Levene’s and Box’s M tests showed no violations of homogeneity of variances or equality of covariance matrices. Shapiro-Wilk tests confirmed normality for learning trials (A1–A5) at both time points. In contrast, deviations from normality were found in the recall conditions. A6 (direct recall) deviated significantly in the IG at t_1_ (*p* = .014) and in both groups at t_2_ (*p* <.05). For A7 (delayed recall), the tauG deviated at t_1_ (*p* = .005), and both groups at t_2_ (*p* <.05). These results indicate moderate normality violations for direct and delayed recall, warranting cautious interpretation.

For VLMT A1–A5, there was no significant time × group interaction, *F*(1, 37) = 1.16, *p* = .288, *η²* = .030. No significant main effects of time, *F*(1, 37) = 1.06, *p* = .309, *η²* = .028, or group, *F*(1, 37) = 0.04, *p* = .853, *η²* = .001, were observed.

For VLMT A6, the analysis also revealed no significant time × group interaction, *F*(1, 37) = 1.50, *p* = .228, *η²* = .039, and no significant main effects of time, *F*(1, 37) = 0.07, *p* = .787, *η²* = .002, or group, *F*(1, 37) = 0.29, *p* = .593, *η²* = .008.

In contrast, the analysis for VLMT A7 revealed a statistically significant time × group interaction, *F*(1, 37) = 8.51, *p* = .006, *η²* = .187, reflecting a large effect size. No significant main effect of time, *F*(1, 37) = 1.27, *p* = .267, *η²* = .033, or group, *F*(1, 37) = 0.09, *p* = .762, *η²* = .003, was found. Descriptive statistics for all VLMT scales are presented in **Table 7a**.

**Table 6:** Summary of VLMT mediation models.

Outcome (Y)	Direct Effect (*c*’)	*SE* (*c*’)	*p* (*c*’)	Indirect Effect (*ab*)	BootSE (*ab*)	BootLLCI	BootULCI	Total Effect (*c*)	*SE* (*c*)	*p* (*c*)
VLMT A1–A5	0.11	0.14	.419	0.00	0.11	-0.22	0.21	0.11	0.09	.242
VLMT A6	-0.03	0.04	.457	0.01	0.03	-0.05	0.08	-0.14	0.03	.652
VLMT A7	-0.03	0.04	.515	0.01	0.03	-0.05	0.08	-0.02	0.02	.529

All mediation analyses were conducted using PROCESS Model 4 with 5,000 bootstrap samples. PTSD symptom severity (PCL, t_1_) was entered as the predictor (X), depressive symptoms (BDI-II, t_2_) as the mediator (M), and various VLMT outcomes (t_2_) as dependent variables (Y). Reported are unstandardized coefficients (*B*), standard errors (*SE*), and *p*-values for the direct (*c*′), indirect (*ab*), and total (*c*) effects. Bootstrap 95% confidence intervals (LLCI, ULCI) are given for the indirect effects.

### 3.5 Visual learning and memory performance

The Shapiro–Wilk test revealed significant deviations from normality for Interference Performance (B) in both groups at both time points, for Short-Delay Recall (A6) in the IG at t_2_, and for the Forgetting Rate in the tauG at t_2_ (*p* < .05). All other variables met the assumption of normality. Levene’s test confirmed homogeneity of variances, except for Long-Delay Recall (A7) at t_1_ (*p* = .023). Sphericity was confirmed for all within-subject comparisons (Mauchly’s *W* = 1.000).

For learning performance (A1–A5), neither main nor interaction effects were significant (*F*(1, 34) = 1.50, *p* = .230, *η*² = .042; interaction: *F*(1, 34) = 2.01, *p* = .165, *η*² = .056), indicating stable performance with small to medium effects.

Interference performance (B) also remained unchanged over time (*F*(1, 34) = 1.01, *p* = .323, *η*² = .029), and the interaction effect was nonsignificant (*F*(1, 34) = 1.48, *p* = .232, *η*² = .042), reflecting small effects.

For short-delay recall (A6), the main effect was nonsignificant (*F*(1, 34) = 0.01, *p* = .945, *η*² = .000), but a marginally significant interaction emerged (*F*(1, 34) = 3.51, *p* = .070, *η*² = .094), suggesting a potential medium effect and differential group development.

Long-delay recall (A7) showed a significant interaction (*F*(1, 34) = 5.46, *p* = .025, *η*² = .138), indicating a medium-to-large group effect with greater gains in the tauG. The main time effect was nonsignificant (*F*(1, 34) = 1.98, *p* = .168, *η*² = .055).

Finally, the forgetting rate (A5–A7) showed a significant main effect of time (F(1, 34) = 8.38, *p* = .007, *η*² = .198), which constitutes a large effect, indicating that forgetting significantly increased across sessions. The group × time interaction showed a statistical trend (F(1, 34) = 3.37, *p* = .075, *η*² = .090), suggesting a medium effect and possible group-specific differences in memory consolidation trajectories. An overview of all interaction, time, and group effects from the repeated-measures ANOVAs for the VLMT and VWLT subscales is presented in **Table 5**.

**Table 5:** Mixed-design ANOVAs results for VLMT and VWLT-subscales: interaction, time, and group effects.

Variable	Interaction *F*	Interaction *p*	Interaction *η*²	Time *F*	Time *p*	Time *η*²	Group *F*	Group *p*	Group *η*²
VLMT A1–A5	1.16	.288	.03	1.06	.309	.03	0.04	.853	.00
VLMT A6	1.50	.228	.04	0.07	.787	.00	0.29	.593	.01
VLMT A7	8.51	.006*	.19	1.27	.267	.03	0.09	.762	.00
VWLT (A1–A5)	2.01	.165	.06	1.49	.230	.04	1.24	.273	.04
VWLT (B)	1.48	.232	.04	1.01	.232	.03	0.80	.377	.02
VWLT (A6)	3.51	.070	.09	0.01	.945	.00	2.99	.093	.08
VWLT (A7)	5.46	.025*	.14	1.98	.168	.06	1.23	.276	.04
VWLT (A5–A7)	3.37	.075	.09	8.38	.007*	.2	0.09	.771	.00

*F*-values, *p*-values, and partial eta squared *(η*²) for interaction, time, and group effects are reported for each VLMT and VWLT-subscales.

### 3.6 Association of depression, PTSD and verbal long-term memory

The following analyses are exploratory and are reported to provide a comprehensive overview of the data; given the limited sample size and statistical power, null findings should be interpreted with caution.

To examine whether depressive symptoms mediate the link between posttraumatic stress and verbal memory, three mediation analyses were run using PROCESS (Model 4, 5,000 bootstraps, 95% CI). PTSD symptoms at baseline (PCL t_1_) served as the predictor (X), depressive symptoms at post-assessment (BDI-II t_2_) as mediator (M), and verbal memory outcomes at post-assessment as dependent variables (Y): verbal learning (VLMT A1–A5), short-delay recall (VLMT A6), and long-delay recall (VLMT A7).

**Figure 3:**
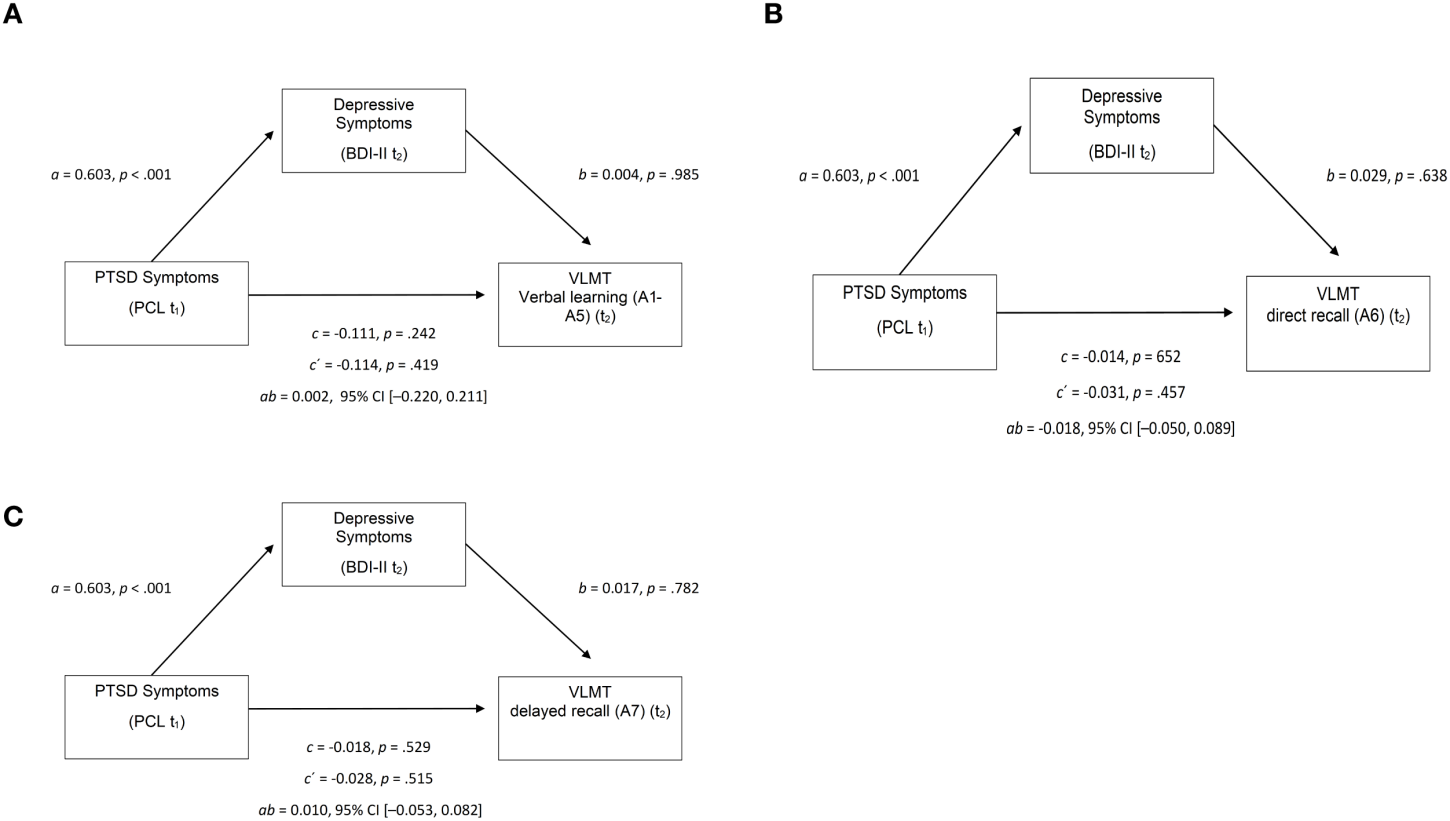
**(a)** Mediation Model: PCL →BDI-II→VLMT Verbal learning (A1-A5). **(b)** Mediation Model: PCL →BDI-II→VLMT direct recall (A6) **(c)** Mediation Model: PCL →BDI-II→VLMT direct recall (A7). PCL, PTSD Checklist (t_1_); BDI-II, Beck Depression Inventory-II (t_2_); VLMT, Verbal Learning and Memory Test. Coefficients represent unstandardized regression weights for paths a, b, and c. CI, confidence interval.

For verbal learning (A1–A5 t_2_), the total effect of PTSD symptoms was nonsignificant, *b* = –0.111, *p* = .242. PTSD symptoms predicted depressive symptoms, *b* = 0.603, *p* <.001, but depressive symptoms did not predict verbal learning, *b* = 0.004, *p* = .985. The indirect effect was nonsignificant, *b* = 0.002, 95% CI [–0.220, 0.211] (see **Figure 3a**).

For direct recall (A6 t_2_), the total effect of PTSD symptoms was nonsignificant, *b* = –0.014, *p* = .652. PTSD symptoms predicted depressive symptoms (*b* = 0.603, *p* <.001), but depressive symptoms did not predict recall (*b* = 0.029, *p* = .638). Neither the direct effect (*b* = –0.031, *p* = .457) nor the indirect effect (*b* = 0.018, 95% CI [–0.050, 0.089]) was significant (see **Figure 3b**).

For delayed recall (A7 t_2_), the total effect of PTSD symptoms was nonsignificant, *b* = – 0.018, *p* = .529 (see **Figure 3c**). PTSD symptoms predicted depressive symptoms (*b* = 0.603, *p* <.001), but depression did not predict delayed recall (*b* = 0.017, *p* = .782). The indirect effect was nonsignificant, *b* = 0.010, 95% CI [–0.053, 0.082].

Across all models, PTSD symptoms predicted higher depressive symptoms, but depression did not significantly predict verbal memory outcomes. Thus, no evidence emerged for depression mediating the relationship between PTSD symptoms and verbal memory performance. Effect estimates are summarized in **Table 6**.

### 3.7 Association of depression, PTSD and visuospatial long-term memory

To test whether depressive symptoms mediate the link between PTSD severity and visuospatial memory, mediation analyses (PROCESS Model 4, 5,000 bootstrap samples) were conducted. PCL (t_1_) served as the predictor, BDI-II (t_2_) as the mediator, and VWLT subscales at t_2_ as outcomes: learning (A1–A5), interference (B), short-delay recall (A6), long-delay recall (A7), and forgetting rate (A5–A7). Across all models, PCL consistently predicted BDI-II (*B* = 0.56, *p* <.001), but neither the direct nor indirect effects on VWLT outcomes were significant, as all 95% confidence intervals included zero. Thus, no evidence emerged for a mediating role of depression in visuospatial memory performance.

**Figure 4:**
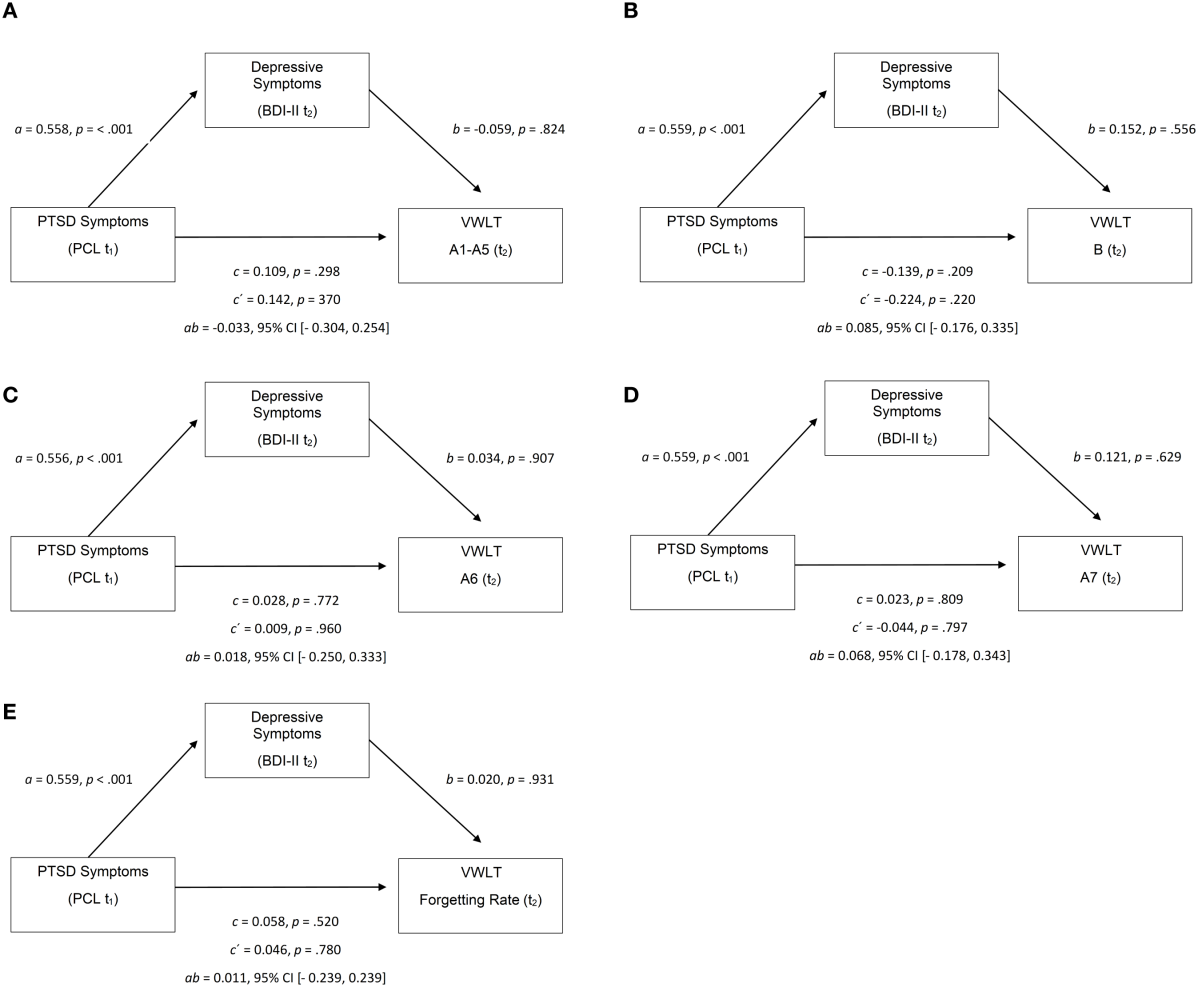
**(a)** Mediation Model: PCL→BDI-II→VWLT A1-A5 **(b)** Mediation Model: PCL→BDI-II→VWLT (B) **(c)** Mediation Model: PCL→BDI-II→VWLT A6 **(d)** Mediation Model: PCL→BDI-II→VWLT A7 **(e)** Mediation Model: PCL→BDI-II→VWLT Forgetting Rate. PCL, PTSD Checklist (t_1_); BDI-II, Beck Depression Inventory-II (t_2_); VWLT, Visual Path Learning Test; A1–A5, learning performance (t_2_). Coefficients represent unstandardized regression weights for paths a, b, and c. CI, confidence interval.

Effect estimates are summarized in **Table 7**, and the model structure is visualized in **Figures 4a-e**, illustrating the consistent model and nonsignificant mediation effects.

**Table 7:** Summary of VWLT mediation models.

Outcome (Y)	Direct Effect (*c*’)	*SE* (*c*’)	*p* (*c*’)	Indirect Effect (*ab*)	BootSE (*ab*)	BootLLCI	BootULCI	Total Effect (*c*)	*SE* (*c*)	*p* (*c*)
Learning Performance (A1–A5)	0.14	0.16	.370	-0.03	0.14	-0.3	0.25	0.11	0.11	.298
Interference Performance (B)	-0.22	0.18	.220	0.08	0.13	-0.18	0.33	-0.14	0.11	.209
Short-Delay Recall (A6)	0.01	0.18	.960	0.02	0.15	-0.25	0.33	0.03	0.09	.772
Long-Delay Recall (A7)	-0.04	0.17	.797	0.07	0.13	-0.18	0.34	0.02	0.1	.809
Forgetting Rate (A5–A7)	0.05	0.16	.780	0.01	0.12	-0.24	0.24	0.06	0.09	.520

All mediation analyses were conducted using PROCESS Model 4 with 5,000 bootstrap samples. PTSD symptom severity (PCL, t_1_) was entered as the predictor (X), depressive symptoms (BDI-II, t_2_) as the mediator (M), and various VWLT outcomes (t_2_) as dependent variables (Y). Reported are unstandardized coefficients (*B*), standard errors (*SE*), and *p*-values for the direct (*c*′), indirect (*ab*), and total (*c*) effects. Bootstrap 95% confidence intervals (LLCI, ULCI) are given for the indirect effects.

## 4 Discussion

The present study investigated whether c-bnt combined with treatment as usual trauma therapy may reduce PTSD and depressive symptoms and improve verbal and visuospatial long-term memory in soldiers with PTSD more than treatment as usual alone. Overall, c-bnt was associated with greater reductions in PTSD symptoms and depressive symptoms at post-treatment compared to the tauG. Post-treatment group differences were most pronounced for the PCL subscales avoidance and negative cognition and for depression severity assessed with the BDI-II.

The observed reduction in avoidance symptoms in the c-bnt group may reflect indirect behavioral and regulatory mechanisms rather than trauma-specific processing. Avoidance represents a behavioral coping strategy that is closely linked to emotional regulation and action readiness and may therefore be particularly sensitive to interventions that promote structured task engagement. The daily, goal-directed nature of c-bnt may have fostered behavioral activation, increased tolerance of cognitive and emotional demands, and enhanced perceived self-efficacy, thereby reducing avoidance tendencies. From this perspective, changes in avoidance may be more readily observable than changes in intrusion or arousal, which are more directly tied to trauma memory processing.

Verbal and visuospatial long-term memory did not improve across the treatment phase in either group; however, in delayed visuospatial recall, the tauG showed greater improvements, a finding that warrants careful consideration given that mediation analyses indicated no indirect effect of depressive symptoms on long-term memory performance. One possible explanation is interference or cognitive overload effects in the intervention group, as the additional daily cognitive training was delivered in parallel with intensive trauma-focused therapy and may have taxed available cognitive resources. Such interference could have selectively affected tasks requiring visuospatial encoding and consolidation. Alternatively, task-specific learning effects may have favored the tauG, as repeated exposure to visuospatial material within the therapeutic context or routine clinical activities may have differentially supported performance on visuospatial recall tasks. Finally, given the exploratory design and limited sample size, these group differences may also reflect unspecific variability rather than systematic intervention effects. This pattern was consistent across verbal and visuospatial memory domains.

Taken together, the present findings suggest that c-bnt may be associated with modest, domain-specific symptom signals, particularly in avoidance and depressive symptoms, when applied as an adjunct to inpatient trauma-focused treatment. However, the clinical relevance of these subscale-level changes remains unclear, particularly in the absence of robust improvements in total PTSD symptom severity. Accordingly, these findings should not be interpreted as evidence of overall treatment efficacy but rather as preliminary indications of domain-specific symptom modulation. Importantly, the present findings should not be interpreted as evidence for the efficacy of c-bnt in enhancing long-term memory performance. No reliable improvements in verbal or visuospatial long-term memory were observed, and any cognitive effects should therefore be considered exploratory rather than indicative of memory enhancement efficacy. These results provide novel insights into the complex interrelations between PTSD and memory dysfunction. Our data may inform the development of innovative strategies for the combined use of trauma therapy and c-bnt in the treatment of soldiers with PTSD.

### 4.1 PTSD and long-term memory

Our results are in accordance with previous studies showing that cognitive training can improve PTSD-related symptoms. Meta-analyses have reported small to moderate symptom reductions after working memory training and interventions targeting attention bias modification in PTSD populations (38, 39). These approaches also led to clinical improvement without robust enhancement of cognitive functions.

However, our results are only partially consistent with previous studies that identified robust verbal and visuospatial memory impairments in PTSD populations (34, 35). For example, Johnsen and Asbjørnsen (29) reported large deficits in verbal memory compared to healthy controls in civilian PTSD samples, and Sep, Geuze (35) documented broad impairments in episodic memory across both clinical (mostly civilian) and preclinical PTSD samples. Our sample, comprising active-duty military personnel undergoing inpatient treatment, did not exhibit clear memory deficits at t_1_ baseline measures. VLMT scores were comparable to respective age norms. Visuospatial memory performance (VWLT) was only slightly below normative age average. Thus, our data do not support a pronounced memory dysfunction in this clinical inpatient sample.

However, direct comparability between civilian and military PTSD samples is limited. Civilian PTSD samples typically show a substantially higher proportion of complex PTSD (cPTSD), which is characterized by a higher prevalence of sequential or prolonged traumatic exposure, more pronounced negative cognitions, dissociative symptoms, and marked impairments in emotion regulation. These features are known to be associated with broader and more persistent cognitive dysfunction, including deficits in memory and executive functioning. Accordingly, differences in long-term memory performance between civilian and military PTSD samples may partly reflect differences in trauma structure and symptom profiles rather than true inconsistencies across studies. In addition, military populations differ from civilian samples with respect to premorbid cognitive functioning and selection processes. Military service involves cognitive, physical, and psychological selection mechanisms, and prospective studies have shown largely preserved neuropsychological functioning in soldiers following deployment, particularly in attention and executive domains (53). Such premorbid cognitive resources may buffer against the development of pronounced long-term memory deficits, even in the presence of trauma exposure, and may further contribute to differences in cognitive profiles between military and civilian PTSD populations. In addition, the nature of military trauma differs in important ways from most civilian traumata. Combat-related trauma is often characterized by repeated exposure and may involve moral or existential conflict; however, the assumption of a simple cumulative dose-response relationship remains controversial. Evidence suggests that symptom severity and cognitive outcomes are not solely determined by the number of deployments, but are strongly influenced by the subjective meaning, intensity, and moral context of traumatic experiences (54). Future studies should therefore investigate how individual predispositions, trauma type, and military-specific experiences interact to shape neurocognitive outcomes in PTSD.

Few studies have specifically investigated training effects on long-term memory in PTSD. Moradi, Moshirpanahi (33) applied Memory Specificity Training in combat veterans and showed improvements in autobiographical memory and reductions in PTSD symptoms. Bomyea, Caudle (32) tested a computerized working memory intervention in U.S. veterans and report symptom reductions. Although participants slightly improved within the training environment due to repeated practice and feedback, these improvements did not generalize to untrained standardized neuropsychological memory tests. Our data are in line with these previous reports in that we also found symptom reduction without robust improvement of verbal and visuospatial long-term memory.

The lack of clear memory enhancement in our sample may at least in part be due to relatively intact long-term memory functions at t_1_ in both groups. Moreover, the relatively short training duration and the high therapeutic load during inpatient trauma treatment as usual may have diluted significant effects on untrained long-term memory tasks in the IG. It should be noted that the c-bnt intervention was not specifically designed to target long-term memory consolidation processes. Any potential effects on verbal or visuospatial long-term memory were considered indirect, for example via general task engagement or attentional demands, rather than through targeted modulation of emotion–cognition integration or memory consolidation processes. Given the intervention duration of three weeks in the absence of targeted consolidation training, measurable changes in standardized long-term memory performance were not hypothesized. In addition, baseline performance on the VLMT and VWLT was largely within the normative range, which may have limited observable improvement due to ceiling effects. The lack of observable transfer from the trained to untrained tasks in our study may suggest that the cognitive resources targeted in the training may not have sufficiently overlapped with those required in standard neuropsychological long-term tests such as the VLMT and VWLT. In addition, the relatively high training intensity of approximately one hour per day, delivered in parallel with intensive inpatient trauma-focused therapy, may have contributed to cognitive fatigue or overload. Trauma-focused treatment places substantial emotional and cognitive demands on patients, and additional daily cognitive training may have taxed available cognitive resources rather than facilitating consolidation. Such overload effects could have attenuated potential benefits of the training on long-term memory performance, particularly in domains requiring sustained encoding and consolidation processes. This highlights a central challenge in cognitive intervention research. For training-induced improvements to generalize, the trained tasks need to engage core processes shared with transfer tasks, a condition that may not have been sufficiently met in the present intervention. Future studies should therefore focus on the degree to which specific cognitive functions trained in each module may concretely lead to neurocognitive improvements in broader, functionally related domains.

### 4.2 Depression

Our finding that depressive symptoms could be reduced by c-bnt, but depression did not mediate memory performance, contrasts with prior evidence linking depression to cognitive dysfunction. Clinical studies and meta-analyses have demonstrated that individuals with depression, especially in clinical and inpatient samples, show consistent impairments in executive functioning (e.g., cognitive flexibility, inhibitory control), attention regulation, and episodic memory, particularly in verbal learning and delayed recall (19, 23, 24). Military-specific studies further indicate that depression is associated with cognitive inefficiencies. In a large veteran sample, depressed individuals exhibited significant impairments in declarative long-term memory and attention, suggesting that memory-related difficulties are also relevant in military populations (55). In addition, neuroscientific research has shown that depression can impair the encoding and retrieval of episodic memories, particularly for positive stimuli, through dysregulation of reward-related neural systems (56).

However, in our sample, the reduction in depressive symptoms after c-bnt did not translate into improved verbal or visuospatial long-term memory. This suggests that depressive symptoms alone do not explain the cognitive deficits commonly found in PTSD, which may instead be rooted in broader neurocognitive dysfunctions. Supporting this view, Scott, Lynch (5) demonstrated that memory and executive abilities independently predicted treatment outcomes in veterans with comorbid PTSD and substance use disorders, highlighting that cognitive functioning itself is a critical factor in determining therapeutic success.

Importantly, we observed symptom reductions in both PTSD and depression, yet memory performance remained mainly unchanged. This finding is consistent with previous research suggesting that cognitive impairments in PTSD, particularly in verbal and visuospatial long-term memory, may be relatively stable and require long and intensive interventions to produce substantial improvements (26, 35). For example, Schweizer, Samimi (38) demonstrated moderate improvements in cognitive control and affect regulation following a 6-week emotional working memory training in adolescents with PTSD. Likewise, Bomyea, Caudle (32) found significant symptom reductions after a 4-week computerized working memory training in U.S. veterans, but only minimal gains on untrained neuropsychological tasks. Polak, Witteveen (26) concluded that executive and memory functions in PTSD are often resistant to change, especially in short-term interventions. Given the data, the relatively brief duration of our c-bnt (three weeks) may have been too short to elicit observable transfer effects on standardized long-term memory measures. The apparent stability of cognitive performance in our sample likely reflects the chronic nature of PTSD-related memory deficits and illustrates the therapeutic challenge of achieving cognitive improvements in chronic cases.

Although our intervention was delivered in parallel with standard trauma-focused inpatient treatment, c-bnt itself did not include trauma-specific therapeutic components such as narrative exposure, imaginative reliving, or cognitive restructuring. Instead, it was a pragmatic and practical cognitive training, consisting of task-oriented training modules targeting attention, planning, and memory through repetitive, emotionally neutral computer-based exercises. Therefore, the observed reductions in avoidance and negative cognitions in the IG may not result from progress in trauma processing per se, but rather from indirect therapeutic mechanisms. Although the training did not explicitly target emotional processing, several task characteristics may nonetheless be relevant for emotion-cognition integration. The c-bnt modules required sustained attention, executive monitoring, and adaptive adjustment to increasing task demands. Such processes are closely linked to cognitive control mechanisms that support emotion regulation under conditions of stress or heightened arousal. From this perspective, c-bnt may have indirectly engaged emotion-cognition integration and coordination processes, providing a plausible, though indirect, mechanistic link to reductions in avoidance and negative cognitions. Empirical findings suggest that emotion-focused cognitive trainings, such as emotional working memory training (44), can enhance affective control and attentional regulation without incorporating trauma-focused psychotherapeutic interventions. For instance, Schweizer, Grahn (44) demonstrated that emotional working memory training improved PTSD symptoms by strengthening top-down control and affect regulation. These effects were attributed to increased cognitive activation and reinforcement of self-regulatory capacities.

Moreover, structured task engagement, such as implemented in our c-bnt modules, may foster a sense of mastery and self-efficacy, psychological resources known to buffer against depressive symptoms and improve psychological functioning (5). Such indirect mechanisms may have contributed to symptom improvements observed in our study in the absence of significant cognitive gains or mediation by depression. Furthermore, the regular structure and positive feedback within the training modules may have acted as behavioral activation elements, which are known to alleviate depressive symptoms. These active components of the applied c-bnt may have fostered self-efficacy in the soldiers. Empirical evidence highlights the importance of perceived self-efficacy in veterans. Blackburn and Owens (57) found that general self-efficacy was significantly associated with lower PTSD and depression severity among veterans. The authors argue that self-efficacy may successfully buffer the impact of combat exposure. In a sample of Iraq/Afghanistan veterans, higher self-efficacy was linked to a lower likelihood of seeking mental health treatment. This finding confirms the important role of self-efficacy for mental health of soldiers in that it demonstrates its link to self-regulatory coping and resilience (58). The structured, mastery-driven nature of c-bnt, characterized by repeated task success and positive feedback, may thus have enhanced self-efficacy and indirectly contributed to depression and PTSD symptom improvement in our study. Benefits in self-efficacy may also explain reduction of depressive symptoms in the absence of statistically significant gains in long-term memory performance and mediating effects of depression. At the same time, because both groups received identical inpatient trauma-focused treatment, additive effects of c-bnt cannot be fully disentangled from nonspecific factors. Increased engagement in structured daily activities, additional cognitive stimulation, and heightened task involvement, reflected in sustained attentional engagement, repeated goal-directed task execution, and continuous performance feedback, may have contributed to symptom reduction independently of specific training effects. Moreover, expectancy effects related to receiving an additional intervention may have influenced outcomes, particularly in the absence of blinding of the study. These nonspecific mechanisms should therefore be considered as potential contributors to the observed effects and warrant more stringent control in future studies. Depressive symptoms and PTSD-related negative cognitions represent closely related, but distinct constructs, which is reflected in their strong conceptual and empirical association across analyses. Future studies should therefore investigate differential effects of specific c-bnt modules and tasks on mediators like self-efficacy.

### 4.3 Scientific and clinical implications

This study has several methodological and conceptual strengths. First, it represents one of the few controlled trials to examine the effects of c-bnt on both clinical symptoms and cognitive functioning in PTSD. While previous research has predominantly addressed single cognitive domains such as working memory or attention (38, 39), our study broadened the focus to include both verbal and visuospatial long-term memory functions. This approach aligns with the growing evidence that PTSD affects multiple memory systems (35) and responds variably to cognitive interventions. Second, the study applied a theory-driven mediational framework, testing whether depressive symptoms could explain potential links between PTSD severity and memory performance. Although the mediation models did not yield significant indirect effects, we found a positive association between PTSD symptom severity at baseline and depressive symptom severity at post-assessment. This relationship supports the assumption of interrelated symptom domains and justifies the application of a mediational framework, even in the absence of a statistically significant mediation effect. Third, the study used well-established, standardized neuropsychological assessments (VLMT, VWLT) to objectively measure cognitive functions. Moreover, both verbal and visuospatial domains were assessed using parallel learning and recall paradigms, allowing for a differentiated analysis of modality-specific effects. Fourth, the study targeted a military population, a group that is both clinically relevant and underrepresented in cognitive training research. PTSD in military contexts often presents chronic trajectories, high functional impairment, and complex comorbidities, making this group an important clinical target for non-trauma-focused interventions. Finally, the study applied a c-bnt that could be implemented flexibly in clinical or occupational settings. This increases the ecological validity of the approach and points to practical applications beyond specialized PTSD clinics. Although the intervention required daily training time in addition to trauma therapy, its standardized, self-guided, and non-trauma-focused format may still offer practical value as a low-threshold adjunct in settings where access to psychotherapy is limited or where patients are reluctant to engage in trauma-focused interventions. The standardized and fully digital format reduces therapist dependency and allows for individualized training schedules, which is particularly relevant for military personnel with irregular schedules or operational deployments. Moreover, by engaging participants in structured, self-directed cognitive exercises, the intervention may foster a sense of self-efficacy and active involvement in the therapeutic process, an aspect that is often limited in traditional trauma-focused treatments, which tend to be more therapist-centered.

### 4.4 Military relevance

The findings of this study suggest that c-bnt may serve as a pragmatic, low-threshold adjunct to trauma-focused therapy in military settings that may contribute to modest, domain-specific symptom improvement in PTSD and depression. Improvement of avoidance and negative cognitions is of particular clinical relevance in military populations, where emotional numbing, avoidance behavior, and functional impairment can compromise mission readiness, interpersonal functioning, and reintegration into social and occupational roles. Moreover, a reduction in avoidance may not only support daily coping but also therapeutic engagement, which is often hindered by stigma, distrust, or fear of reactivation in active-duty personnel.

For soldiers who refuse psychotherapy at all or do not want to engage in trauma-focused treatment approaches, c-bnt may offer a preparatory strategy of stabilization. By activating cognitive processes through emotionally neutral and mastery-oriented tasks, it may reduce arousal and foster psychological readiness for subsequent trauma processing. Given that access to psychotherapy is often limited due to deployment schedules, geographic dispersion, or resource constraints, the fully digital and self-guided structure of c-bnt makes it particularly suitable for integration into military health services. Modular designs of c-bnt allows flexible use across different phases of the deployment cycle, including pre-deployment preparation, mental health support during deployment, and post-deployment reintegration.Future studies should focus on the optimization of training duration and training tasks and identify most appropriate time points and subgroups for c-bnt. In this context, trauma chronicity, comorbidities, and prior treatment history are likely to be key factors. Moreover, studies embedded in real-world military settings are needed to test the feasibility, acceptability, and impact of c-bnt under operational conditions.

## 5 Limitations

Despite these strengths, several limitations of this study should be considered. The relatively high rate of termination of study participation ahead of time from baseline to post-assessment (35.85%) represents an important limitation and may have reduced statistical power, particularly for interaction and mediation effects. This rate is primarily attributable to organizational and treatment-related factors inherent to inpatient military care, such as early discharge or service-related constraints, rather than study-related burden. In addition, attrition was influenced by military-specific operational demands, including short-term missions, intensified training exercises, courses, and command-related assignments, which have increased substantially in recent years and further limited participant availability. Nevertheless, the reduced sample size at post-assessment limits the robustness and statistical conclusion validity of the findings. Results should therefore be interpreted with caution. Given the limited sample size and the primarily organizational reasons for dropout, systematic comparisons between completers and non-completers were not conducted. The presence of dropout-related bias can thus not be fully ruled out.

Moreover, treatment as usual was delivered by several therapists using different trauma-focused therapeutic approaches, and specific treatment modalities were not documented at the individual level. Although patients were randomly assigned to therapists as part of routine clinical care, and the same pool of therapists treated both groups, therapist effects and differences in therapeutic approaches could not be controlled.

In addition, the quasi-randomized design does not allow full control for expectancy effects or baseline differences in psychological resilience between groups. This may have contributed to observed group differences. Furthermore, the intervention group had a significantly higher number of deployments at baseline, which may perhaps reflect greater cumulative trauma exposure and potentially different resilience profiles. This imbalance may have biased the results in either direction: greater trauma exposure may be associated with more severe baseline symptomatology and thus greater potential for symptom reduction, whereas higher resilience or prior operational experience may have facilitated engagement with the cognitive training. However, the number of deployments represents only a coarse proxy for trauma exposure and does not necessarily capture the intensity, severity, or subjective burden of traumatic experiences. Accordingly, a small number of deployments involving highly critical or morally injurious events may result in greater psychological strain than multiple deployments with comparatively moderate exposure. As the number of deployments was not controlled for in the primary analyses, its potential influence on the observed effects cannot be ruled out.

Moreover, the therapeutic alliance was not formally assessed and therefore could not be included in the analyses. It needs to be considered that the sample size was rather small, which limits statistical power to detect small or interaction effects, particularly in mediation analyses. Although the study was based on an *a priori* sample size calculation, the dropout rate reduced the available sample at post-assessment, thereby limiting statistical power, particularly for detecting small effects and for interaction and mediation analyses. Accordingly, null findings should be interpreted with caution. In particular, the available sample size was insufficient to reliably detect modest cognitive effects or interaction effects in mixed-model analyses. The mediation analyses should therefore be considered exploratory and hypothesis-generating. The study design does not support assumptions about temporal or causal relationships. Accordingly, both indirect and null effects must be interpreted with strong caution. Violations of statistical assumptions (e.g., normality and homogeneity of variance) observed for some outcome measures further reduce the robustness of the statistical inferences and limit the strength of the conclusions that can be drawn.

The intervention period was relatively short and may not have been sufficient to induce significant improvements in long-term memory performance. Memory functions, particularly in PTSD, may require longer and more intensive training to show reliable changes. Finally, the clinical outcomes were assessed via self-report measures (PCL, BDI-II). Although these instruments are well validated standards, the inclusion of clinician-administered diagnostic interviews (e.g., CAPS-5) would have strengthened the clinical assessment and confirmed symptom change more objectively.

## 6 Conclusion

In conclusion, this study provides preliminary evidence for modest, domain-specific symptom signals associated with c-bnt, particularly in avoidance and depressive symptoms in military personnel. The observed symptom improvements, particularly in avoidance and negative cognitions, highlight the potential of cognitive interventions as a low-threshold, non-trauma-focused approach for individuals with PTSD. C-bnt did not lead to significant improvements in verbal and visuospatial long-term memory performance, suggesting that symptom relief and cognitive rehabilitation may follow distinct pathways. Beyond its clinical effects, c-bnt may promote a sense of self-efficacy by actively involving patients in structured cognitive exercises, thereby strengthening their perceived control and participation within the therapeutic process. This mechanism could represent an additional pathway through which cognitive training contributes to symptom improvement. These findings underscore the need for more detailed future studies to clarify the mechanisms underlying symptom reduction and cognitive improvement in individuals with PTSD. Note, c-bnt may complement trauma-focused therapy, but it should not be viewed as a substitute for established trauma treatment. Rather, it may serve as an accessible adjunct for symptom management and cognitive activation, particularly in military contexts where flexible and self-guided treatment options are needed.

## Data Availability

The raw data supporting the conclusions of this article will be made available by the authors, without undue reservation.
